# Sequence Analysis of Long-Term Readmissions among High-Impact Users of Cerebrovascular Patients

**DOI:** 10.1155/2017/7062146

**Published:** 2017-05-16

**Authors:** Ahsan Rao, Alex Bottle, Ara Darzi, Paul Aylin

**Affiliations:** ^1^Faculty of Medicine, Dr Foster Unit, Imperial College London, 3 Dorset Rise, London EC4Y 8EN, UK; ^2^Faculty of Medicine, Institute of Global Health, Imperial College London, St Mary's Hospital, Praed Street, London W2 1NY, UK

## Abstract

**Objective:**

Understanding the chronological order of the causes of readmissions may help us assess any repeated chain of events among high-impact users, those with high readmission rate. We aim to perform sequence analysis of administrative data to identify distinct sequences of emergency readmissions among the high-impact users.

**Methods:**

A retrospective cohort of all cerebrovascular patients identified through national administrative data and followed for 4 years.

**Results:**

Common discriminating subsequences in chronic high-impact users (*n* = 2863) of ischaemic stroke (*n* = 34208) were “urological conditions-chest infection,” “chest infection-urological conditions,” “injury-urological conditions,” “chest infection-ambulatory condition,” and “ambulatory condition-chest infection” (*p* < 0.01). Among TIA patients (*n* = 20549), common discriminating (*p* < 0.01) subsequences among chronic high-impact users were “injury-urological conditions,” “urological conditions-chest infection,” “urological conditions-injury,” “ambulatory condition-urological conditions,” and “ambulatory condition-chest infection.” Among the chronic high-impact group of intracranial haemorrhage (*n* = 2605) common discriminating subsequences (*p* < 0.01) were “dementia-injury,” “chest infection-dementia,” “dementia-dementia-injury,” “dementia-urine infection,” and “injury-urine infection.”* Conclusion*. Although common causes of readmission are the same in different subgroups, the high-impact users had a higher proportion of patients with distinct common sequences of multiple readmissions as identified by the sequence analysis. Most of these causes are potentially preventable and can be avoided in the community.

**Conclusion:**

Although common causes of readmission are the same in different subgroups, the high-impact users had a higher proportion of patients with distinct common sequences of multiple readmissions as identified by the sequence analysis. Most of these causes are potentially preventable and can be avoided in the community.

## 1. Introduction

Emergency hospitalisation is distressing and physically challenging for a patient. It bears a psychological impact on a patient because changes in the environment can lead to disorientation, delirium, and further decline in one's health status [1]. It directly affects the health status of the patient regardless of the cause of admission even if the patient had completely recovered or did not face any adverse events [2]. It was shown that 75% of the elderly patients hospitalised did not return to their baseline functional status and 15% of them were discharged to a nursing home [3]. Emergency hospitalisation has been associated with almost one-third of the cost incurred by the health system [4]. Health trusts and policy makers have placed various interventions to prevent unplanned readmissions to the hospital [5]. Despite various efforts, the rate of readmission continues to rise [6].

In the US, hospitals are penalised and their payments are not reimbursed if the observed readmission rate is higher than expected under the hospital readmissions reduction program (HRRP) since year 2010 [7]. Previous studies have tried to identify causes of emergency readmissions so that appropriate measures are placed in the community [8]. For example, infections, recurrent stroke, and cardiovascular conditions have been shown to be the common causes of short-term readmission in stroke patients [9]. Hospital administrative data has become a vital tool to assess the main diagnosis for readmission as the information is routinely recorded with good accuracy [10]. The sensitivity of correct diagnosis with the use of discharge diagnosis coding is more than 80% [10].

The high-impact users are a small subgroup of the patient population that have a significantly higher rate of unplanned hospitalisations [11]. They are defined as those patients who have had 3 or more emergency hospitalisations in a year [11]. They are shown to utilise as much as two-thirds of the healthcare resources [12]. Risk profiling of the patient population to identify these patients provides health policy makers an opportunity to plan optimum and individualised patient care by allocating appropriate resources, analysing trends in the health status of a population, and finding the risk factors that can be modified to prevent decline in the health status at a population level [13]. It will be interesting to further evaluate causes of emergency readmissions in the high-impact group and to assess if the causes of emergency readmissions differ from those of the low-impact users.

Understanding the chronological order and the sequence of the causes of readmissions may help us to assess any repeated chain of events for high hospital care use by high-impact users. Previous studies have shown that the temporal order of events has significant impact on the outcomes of patients [14]. For example, incidence of heart failure following atrial fibrillation was shown to be associated with high mortality compared with those patients who were diagnosed with atrial fibrillation after heart failure [15]. Similarly, a particular sequence of vaccination, that is, measles vaccination followed by DTP (diphtheria-tetanus-pertussis) or IPV (inactivated polio vaccine), was an important determinant of high mortality rate in female infants [16]. So far, it is still not certain whether distinct and common sequences of causes of readmissions are associated with increased hospital use.

Sequence analysis is a well-established technique in social science and criminology that assess the chronological order of events among subgroups with different personalities and social behaviour [17]. It had been used to study population behaviour and assess sequences of events related to adverse outcomes. It has the ability to cluster and visualise common sequences of events in the population [18]. Statistical analysis can be used to identify distinct subsequences in a large sequence of events related to a particular subgroup of interest [18]. We have not found any study that have applied sequence analysis to hospital administrative data to assess temporal sequence of events associated with readmissions in the high-impact users. The studies evaluating causes of readmissions with the use of hospital administrative data had merely searched for the common causes of readmission [9]. We hypothesise that sequence analysis can be applied to hospital administrative data to assess the chronological order of the causes of readmissions. We focused on the causes of emergency readmissions to ascertain common chains of repeated events associated with high use of hospital care. Hence, if a common pattern of readmissions is recognised, it can help with cost-effective prevention of these events in the community. For the purpose of the analysis, we chose a cohort of cerebrovascular conditions, that is, ischaemic stroke, TIA (transient ischaemic attack), and nontraumatic intracranial haemorrhage, because the conditions usually have a discrete acute onset with initial treatment in the hospital [19]. These conditions are associated with high population morbidity [19]. The rate of readmissions is high and patients incur a huge cost on health systems worldwide [20].

## 2. Methods

### 2.1. Database

Data from Hospital Episode Statistics (HES) were used for this retrospective cohort study. It is the annual patient administrative data collected by the Department of Health in England. It covers information on all inpatient hospital stays in public National Hospital Service (NHS) hospitals in the country as well as information on private patients treated in these hospitals [21]. All emergency cases are admitted and initially treated in these hospitals [21]. Each hospital admission is recorded as a “spell” consisting of a number of “consultant episodes,” which denotes period of care under different consultants during their hospital admission [22]. If the patient admission includes transfer to other hospitals before they are discharged, the whole period of care is recorded as a “superspell.” For each patient, information from their superspell was obtained, such as primary diagnosis, number of secondary diagnoses, primary operation, admission date, discharge date, length of stay, discharge destination, and admission source. The primary diagnosis is the one that utilised most of the hospital resources. The primary diagnosis and the list of the secondary diagnosis are recorded using ICD-10 classification, whereas Office of Population, Census, and Survey version 4.7 (OPCS 4.7) coding is used for primary and secondary procedures. Each patient has a unique anonymised HES identifier that was used to recognise further hospital episodes. The data only contains information on mortality for the patients who died in the hospital. It does not include information on the date of death for the patients who died in the community.

### 2.2. Study Population

All adult patients over the age of 18 who had emergency admission for cerebrovascular conditions in the financial year 2010/11 were included in the study. The patient cohort is comprised of all kinds of cerebrovascular conditions, haemorrhagic and ischaemic. The subcategories of nontraumatic intracranial haemorrhage were intracerebral haemorrhage (ICH), subarachnoid haemorrhage (SAH), subdural haemorrhage (SDH), and extradural haemorrhage (EDH). The patients who died during the index admission for stroke were excluded from the analysis. Specific ICD-10 codes (International Classification of Diseases) were used to identify stroke patients using similar codes to those from previous studies: ischaemic stroke (I63x), TIA (G45x, H34x), and nontraumatic intracranial haemorrhage (SAH [I60x], ICH [I61x], and other nontraumatic intracranial haemorrhage including subdural and extradural haemorrhage [I62x]) [23–26]. The sensitivity and specificity of the coding of primary diagnosis using administrative had shown to be higher than 80% and comparable to the diagnosis obtained from clinical data [10]. The patients were followed up for at least 4 years. The data were retrieved for each patient every time they were admitted to a hospital for any reason. Once the cerebrovascular patients were identified, the previous 10 years of HES data were examined to identify any history of previous stroke event admitted to an English NHS hospital. Patients with the history of stroke were retained in the data analysis and previous stroke was used as a risk factor to assess association with subgroups.

The increased use of hospital care in a year was defined as having 3 or more unplanned hospital admissions or having spent more than 46 days in the hospital. The patients were categorised into 3 groups: low-impact users, short-term high-impact users, and chronic high-impact users. The patients who had persistently increased hospital care use every year during the follow-up period were classed as high-impact users. This group also included those patients who died during the follow-up but had high hospital use in the preceding years of the follow-up. The low-impact users were the patients with no increased hospital care use every year during the follow-up period. The remaining population consisted of the patients with at least one year of high hospital care use but not persistent throughout the follow-up period. These patients were labelled as short-term high-impact users.

Previous studies have only included 3 or more unplanned inpatient hospital admissions within a year as a high use of hospital care [11, 27, 28]. We have also included cumulative LOS (cLOS) within a year to include the patients who had less than 3 hospital admissions but spent a significant amount of time in the hospital [29, 30]. The cut-off value for cumulative LOS (cLOS) within a year that accounts for high use has varied in different studies [30]. It is usually chosen as average LOS or the lowest value for 75th and 90th percentile for group's cLOS. In this study, the average cLOS for patients with 3 or more hospital admissions was used as a mark to select cLOS to define high-impact users.

### 2.3. Statistical Analysis

SAS 9.4 software was used to perform descriptive statistics and categorise the patients based on the hospital care use. Information on the comorbidities was obtained from the list of secondary diagnosis for each hospital admission. The population weighted quintiles of the Carstairs deprivation score were used to classify patients according to their neighbourhood deprivation levels [31]. The quintile ranges from 1 to 6, where score of 5 defined the most deprived residences and 6 means not known (missing postcode). The Charlson score was used to calculate the comorbidity burden associated with each patient [32]. Higher score was associated with the severity of comorbidity. The Charlson score for the admission of stroke was obtained from the sum of score of the past medical problems as listed in the previous study [32].

The sequence analysis was conducted using TraMineR package (version 1.8-12) in R language statistical software [18]. The package has the ability to search, identify, and visualise sequences of categorical data. It can analyse large length of sequences of various events. It summarises and displays common sequences in the subgroups. Chi-square test was used to compare the number of subsequences in the subgroups. It was also used to compare categorical variables between the groups. ANOVA was used to assess differences among groups for continuous variables.

All the causes of emergency readmissions for the patient cohort were examined and the common ones that constituted more than 90% of the causes of readmissions were identified. They were categorised into the following groups: gastrointestinal infections, chest infection, gastrointestinal bleeding, ambulatory conditions (heart failure, COPD, asthma, and diabetes), delirium, dementia, epilepsy/seizures, external injuries, nontraumatic intracerebral haemorrhage, Iatrogenic complications, other infections/inflammation of internal organs/skin, cerebrovascular conditions, ischaemic heart disease, nutritional and metabolic disorders, pulmonary embolism and DVT, peripheral vascular disease, and urine infection and urological conditions. There is no standard methodology to categorise causes of readmissions. The classification in this study was adopted from previous studies [9, 26, 33]. All the common conditions causing emergency readmission were identified and then they were grouped based on related pathologies and similar management pathways so that a single modifiable factor in the treatment algorithm can be identified to avoid the category of readmissions. For example, the ambulatory conditions consisted of diseases known to have chronic insidious background and acute flare-ups. Common occurrence of all these conditions would indicate poor secondary prevention of the disease. Similarly, all types of dementia were grouped together in the category “dementia.” The ICD codes for primary causes of nonelective admissions were used to recognise the conditions (supplementary file in Supplementary Material available online at https://doi.org/10.1155/2017/7062146).

## 3. Results

**Figure 1:**
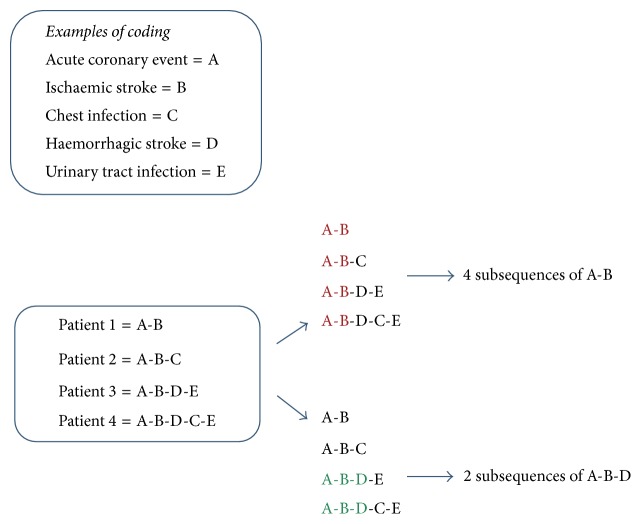
Example of mining of sequence data to identify common subsequences in patient population.

Each category of readmission was coded with a unique alphabet. For each patient, a string of sequence of alphabets was created based on the chronological order of categories of readmissions (Figure 1).

### 3.1. Ischaemic Stroke

**Figure 2:**
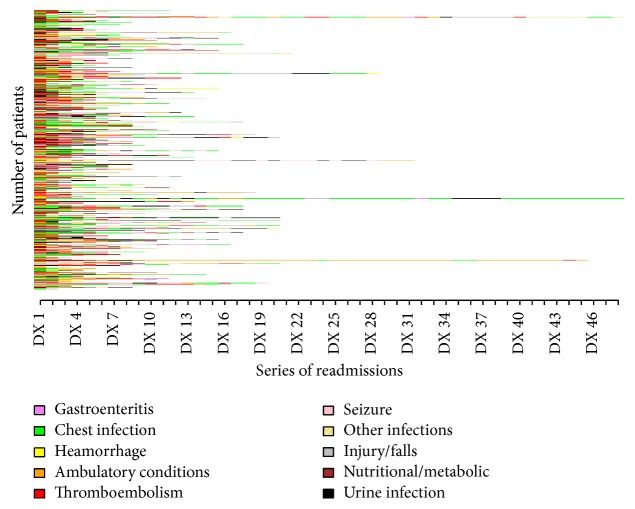
Depiction of ischaemic stroke patients and their colour-coded sequences of readmissions.

The patient population (*n* = 34208) consisted of 48% males and 52% females, with the mean age of 72.2 (SD 13.47). The mean LOS was 15.35 days (SD 22.47) and 14% of the patients lived alone. During the follow-up, 1993 patients died. Of the total patient population, 57.9% of the patients had emergency readmissions. The patient characteristics associated with each group are described in Table 1. The colour-coded sequences of all the patients who had emergency readmissions are displayed in Figure 2. The 5 most common causes of admissions for 4 years' follow-up period were chest infection (19.29%), urine infection and urological conditions (14.74%), external injuries (12.40%), ambulatory conditions (10.77%), and ischaemic stroke (10.14%). The common causes of emergency readmission for each year follow-up period are mentioned in Table 2. Among the low-impact group (*n* = 25758), the 5 most common causes of readmission were chest infection (19.03%), external injuries (14.30%), ischaemic stroke (12.18%), urine infection and urological conditions (13.25%), and ambulatory conditions (9.61%). Among the short-term high-impact group (*n* = 7269), the 5 most common causes of readmission were chest infection (19.00%), urine infection and urological conditions (15.93%), external injuries (11.88%), ambulatory conditions (10.23%), and ischaemic stroke (9.75%). Among the chronic high-impact group (*n* = 1181), the 5 most common causes of readmission were chest infection (21.33%), ambulatory conditions (16.39%), urine infection and urological conditions (13.20%), external injuries (9.69%), and ischaemic stroke (6.44%). Most common discriminating subsequences were identified between groups as described in Table 3. Cumulatively, 39.0% of the chronic high-impact users had 5 most common subsequences compared to 6.6% and 34.4% of the low-impact and short-term high-impact users, respectively. Similarly, comparison was made between common subsequences in the subgroups of high-impact users (Table 4). The 5 most common subsequences were present in 22.5% of the chronic high-impact users as compared to 19.6% of the short-term high-impact users. The 4-year mortality rate was highest among chronic high-impact users (*n* = 751, 63.5%) as compared to low-impact (*n* = 1226, 4.7%) and short-term high-impact (*n* = 19, 0.3%) groups (*p* < 0.001).

Among the patients who survived, the common causes of emergency readmissions were delirium (*n* = 8115, 32.7%), ambulatory conditions (*n* = 5413, 21.8%), cardiac arrhythmia (*n* = 4222, 17.0%), respiratory infections (*n* = 2612, 10.5%), and dementia (*n* = 2004, 8.1%). The common subsequences among these patients were “delirium-cardiac arrhythmia” (*n* = 4003, 16.1%), “cardiac arrhythmia-delirium” (*n* = 3451, 13.9%), “ambulatory conditions-delirium” (*n* = 3257, 13.1%), “delirium-ambulatory conditions” (*n* = 2865, 11.6%), and “delirium-delirium-cardiac arrhythmia” (*n* = 1998, 8.1%). On the other hand, the common causes of readmission among those patients who died during the follow-up were delirium (*n* = 418, 26.7%), cardiac arrhythmia (*n* = 414, 26.4%), ambulatory conditions (*n* = 363, 23.2%), dementia (*n* = 134, 8.6%), and gastrointestinal infections (*n* = 82, 5.2%). The common subsequences of readmissions were “delirium-cardiac arrhythmia” (*n* = 189, 12.1%), “cardiac arrhythmia-delirium” (*n* = 125, 7.9%), “delirium-delirium-cardiac arrhythmia” (*n* = 109, 6.9%), “delirium-ambulatory conditions” (*n* = 96, 6.1%), and “ambulatory conditions-delirium” (*n* = 93, 5.9%).

### 3.2. Intracranial Haemorrhage

The patient population (*n* = 2605) consisted of 63% males and 37% females, with the mean age of 72.25 (SD 13.63). The mean LOS was 10.82 (SD 17.62) and 10.9% of the patients lived alone. During the follow-up, 145 patients died. Of the total patient population, 54.24% of the patients had emergency readmissions. The patient characteristics associated with each group are described in Table 1. Of the emergency readmissions (*n* = 2674), the 5 most common causes of admissions were chest infection (20.50%), external injuries (15.22%), urine infection and urological conditions (13.56%), ambulatory conditions (7.81%), and dementia (6.12%). The common causes of emergency readmission for each year follow-up period are mentioned in Table 2. Among the low-impact group (*n* = 1357), the 5 most common causes of readmission were chest infection (21.64%), external injuries (17.39%), urine infection and urological conditions (12.75%), ambulatory conditions (8.12%), and ischaemic stroke (5.94%). Among the short-term high-impact group (*n* = 1143), the 5 most common causes of readmission were chest infection (19.77%), external injuries (12.78%), urine infection and urological conditions (14.42%), ambulatory conditions (7.80%), and epilepsy/seizure (6.81%). Among the chronic high-impact group (*n* = 174), the 5 most common causes of readmission were chest infection (20.90%), external injuries (12.31%), dementia (11.92%), urine infection and urological conditions (11.62%), and bleeding complications (8.59%). Most common discriminating subsequences were identified between groups as described in Table 3. Cumulatively, 38.4% of the chronic high-impact users had 5 most common subsequences compared to 4.0% and 22.3% of the low-impact and short-term high-impact users, respectively. Similarly, comparison was made between common subsequences in the subgroups of high-impact users (Table 4). The 5 most common subsequences were present in 12.5% of the chronic high-impact users as compared to 12.0% of the short-term high-impact users. The 4-year mortality rate was highest among chronic high-impact users (*n* = 53, 58.9%) as compared to low-impact (*n* = 92, 4.7%) and short-term high-impact (*n* = 0) groups (*p* < 0.001).

Among the patients who survived, the common causes of emergency readmissions were delirium (*n* = 579, 31.4%), ambulatory conditions (*n* = 338, 18.3%), cardiac arrhythmias (*n* = 290, 15.7%), dementia (*n* = 202, 10.9%), and chest infection (*n* = 180, 9.8%). The common subsequences among these patients were “delirium-cardiac arrhythmia” (*n* = 275, 14.9%), “cardiac arrhythmia-delirium” (*n* = 227, 12.3%), “ambulatory conditions-delirium” (*n* = 204, 11.1%), “delirium-ambulatory conditions” (*n* = 175, 9.5%), and “dementia-delirium” (*n* = 144, 7.8%). On the other hand, the common causes of readmission among those patients who died during the follow-up were cardiac arrhythmia (*n* = 40, 33.6%), delirium (*n* = 29, 24.4%), ambulatory conditions (*n* = 16, 13.4%), gastrointestinal infections (*n* = 14, 11.7%), and dementia (*n* = 9, 7.5%). The common subsequences of readmissions were “delirium-cardiac arrhythmia” (*n* = 13, 10.9%), “cardiac arrhythmia-delirium” (*n* = 8, 6.7%), “ambulatory conditions-delirium” (*n* = 7, 5.8%), “delirium-delirium-cardiac arrhythmia” (*n* = 7, 5.9%), and “delirium-dementia” (*n* = 7, 5.9%).

### 3.3. Transient Ischaemic Attack (TIA)

The patient population (*n* = 20549) consisted of 49% males and 51% females, with the mean age of 72.25 (SD 13.63). The mean LOS was 2.96 (SD 6.12) and 10.9% of the patients lived alone. During the follow-up, 945 patients died. Of the total patient population, 58.21% of the patients had emergency readmissions. The patient characteristics associated with each group is described in Table 1. Of the emergency admissions (*n* = 21917), the 5 most common causes of admissions were chest infection (18.73%), urine infection and urological conditions (13.86%), external injuries (13.39%), ambulatory condition (11.67%), and ischaemic stroke (9.87%). Among low-impact users (*n* = 11380), the common causes of emergency readmission for each year follow-up period are mentioned in Table 2. The 5 most common causes among low-impact users were chest infection (16.59%), external injuries (14.72%), ischaemic stroke (12.21%), urine infection and urological conditions (11.54%), and ambulatory conditions (11.17%). The 5 most common causes among short-term high-impact users (*n* = 9285) were chest infection (18.59%), urinary traction infection (15.31%), external injuries (13.02%), ambulatory conditions (11.52%), and ischaemic stroke (9.15%). The 5 most causes among chronic high-impact users (*n* = 1252) were chest infection (25.93%), ambulatory conditions (13.98%), urine infection and urological conditions (13.33%), external injuries (11.37%), and ischaemic stroke (6.65%). Most common discriminating subsequences were identified between groups as described in Table 3. Cumulatively, 40.6% of the chronic high-impact users had 5 most common subsequences compared to 6.9% and 36.5% of the low-impact and short-term high-impact users, respectively. Similarly, comparison was made between common subsequences in the subgroups of high-impact users (Table 4). The 5 most common subsequences were present in 32.9% of the chronic high-impact users as compared to 6.9% of the short-term high-impact users. The 4-year mortality rate was highest among chronic high-impact users (*n* = 40, 62.1%) as compared to low-impact (*n* = 530, 3.4%) and short-term high-impact (*n* = 13, 0.3%) groups (*p* < 0.001).

Among the patients who survived, the common causes of emergency readmissions were delirium (*n* = 5423, 35.4%), ambulatory conditions (*n* = 2891, 18.9%), cardiac arrhythmia (*n* = 2495, 16.3%), respiratory infections (*n* = 1801, 11.8%), and dementia (*n* = 1167, 7.6%). The common subsequences among these patients were “delirium-cardiac arrhythmia” (*n* = 2496, 16.3%), “cardiac arrhythmia-delirium” (*n* = 2158, 14.1%), “ambulatory conditions-delirium” (*n* = 2150, 14.1%), “delirium-ambulatory conditions” (*n* = 1985, 12.9%), and “delirium-delirium-cardiac arrhythmia” (*n* = 1342, 8.8%). On the other hand, the common causes of readmission among those patients who died during the follow-up were cardiac arrhythmia (*n* = 236, 29.5%), delirium (*n* = 208, 26.0%), ambulatory conditions (*n* = 142, 17.7%), dementia (*n* = 85, 10.6%), and gastrointestinal infections (*n* = 51, 6.3%). The common subsequences of readmissions were “delirium-cardiac arrhythmia” (*n* = 88, 11.0%), “cardiac arrhythmia-delirium” (*n* = 69, 8.6%), “delirium-ambulatory conditions” (*n* = 62, 7.7%), “ambulatory conditions-cardiac arrhythmia” (*n* = 59, 7.4%), and “delirium-delirium-cardiac arrhythmia” (*n* = 51, 6.4%).

## 4. Discussion

Common causes and subsequences of emergency readmissions were identified in each subgroup of patients. In general, recurrent ischaemic stroke, external injuries, ambulatory conditions, chest, and urine infections/urological conditions were the common causes of readmissions in all groups of cerebrovascular patients during 4-year follow-up period. Distinct subsequences of readmissions were recognised among the high-impact users. The sequence of readmissions among the high-impact users of ischaemic stroke and TIA mainly consisted of chest infection and urine infection/urological conditions, external injuries, and ambulatory conditions, whereas high-impact users of haemorrhagic stroke with multiple readmissions had common occurrence of chest infection, external injuries, dementia, and seizures.

The common causes of short-term readmissions in cerebrovascular patients identified earlier were similar to the causes observed in long-term follow-up in the study [33, 34]. Previous studies, mainly focusing on ischaemic stroke patients, found recurrent stroke, infections, fractures/falls, and cardiovascular conditions to be common causes of readmissions [9]. Diabetic complications and readmissions due to heart failure were also common in the patient cohort [35]. We also acknowledged readmissions due to ambulatory conditions to be common in cerebrovascular patients. Dementia was more common cause of readmission among patients with intracranial haemorrhage. Ischaemic stroke patients were also readmitted for dementia, especially those who survived during the follow-up period, but it was not one of the top 5 conditions. As noted in earlier studies, the risk of poststroke dementia is high during the first year [18, 36, 37]. The patients of intracranial haemorrhage had higher proportion of those with preexisting dementia and cognitive impairment and those who have undergone intracranial procedure. These factors are all known to increase the risk of dementia after stroke [36–38]. Moreover, generalised symptoms are more common in haemorrhagic stroke, such as nausea, headache, memory loss, and conscious level alteration [39]. This may indicate change in intracranial pressure particularly after someone who had undergone intracranial procedure. Hence, patients are more likely to be admitted for possible dementia following intracranial haemorrhage.

The information of pattern of causes of multiple readmission can help clinician in discharge planning and improve transition of care from secondary to primary care. Of all the conditions that can cause emergency readmissions, the high-impact users suffer from few conditions repeatedly. The high-impact users had a significantly higher proportion of patients with distinct chronological orders of multiple emergency readmissions. Moreover, chronic high-impact users had distinct common subsequences of emergency readmissions as compared to short-term high-impact users. Approximately, 40% of the chronic high-impact users had distinguishable common subsequences as compared to less than 10% of the low-impact users in all cerebrovascular conditions. The knowledge of the sequence of readmissions can help clinicians tailor discharge planning according to the risk of the patient for having multiple readmissions. After ischaemic stroke and TIA event, distinct sequences of multiple readmissions consist of exacerbation of ambulatory conditions and infections. Among the patients with intracranial haemorrhage, patients with multiple readmission often suffer from external injuries, dementia, epilepsy, and urological conditions. Clinicians looking after the patients in the hospital can set up early outpatient review for patients with ambulatory comorbidities. They can also liaise with primary care team for community visits, recommendation of flu vaccinations, regular diabetic checks, and community rehabilitation programs to improve secondary prevention of these conditions. Despite these measures, if a patient gets a readmission for any of these conditions, the clinician can anticipate next likely diagnosis for emergency readmissions and can take steps according to patient's circumstances to prevent those conditions from occurring.

The patients who died during the follow-up period had increased readmission for gastrointestinal infections as compared to those who survived. This included infection from* Clostridium difficile*, which is commonly caused by the inappropriate use of antibiotics. The prevention of gastrointestinal infections can be a significant factor in the reduction of mortality among patients with cerebrovascular conditions, especially the prevention of* Clostridium difficile* infections in the community. This may warrant the same rigorous approach to prevent this infection by appropriate antibiotic prescription in the community as in the hospital in the recent years. Other common causes of emergency admissions and sequences of readmissions were similar in both groups. However, the chronic high-impact users had significantly higher mortality compared to the other subgroups. They had distinct sequences of readmissions which suggest that patients who had multiple readmissions before death were different from those who died shortly after stroke.

In the recent years, the sequence analysis of events has been used in medical research [14]. A particular sequence of resolution of symptoms in pneumonia was associated with poor prognosis [14]. The common sequence with resolution of temperature and abnormal blood pressure occurring first was associated with the high mortality group. Previous studies have used clinical data for sequence analysis. Our use of hospital administrative data in this study is novel. Since primary diagnosis coding for inpatient emergency admissions is routinely recorded, the sample size is large, and there is often national coverage, hospital administrative data provides a good vehicle for sequence analysis [10].

It is crucial to make an effort to identify distinct sequence of causes of emergency readmissions. Although it is hard to define readmission that is potentially preventable, previous studies suggest all emergency readmissions are potentially avoidable, especially those that are due to medical conditions rather than surgical [40]. Our analysis suggested that common subsequences of readmissions in high-impact group mainly contained medical conditions. From the hospital administrative data, it is not possible to isolate information on community factors associated with cause of admissions, for example, whether or not the urinary infection was catheter related. However, it can provide enough information to concentrate on certain conditions that lead to further readmissions like chest infection as shown in our data. An elderly patient with multiple comorbidities has few body reserves to combat complications [41]. An acute medical condition leading to hospital admission deteriorates functional health of the patient [41]. Once the patient is discharged from the hospital, his health status does not fully recuperate and he becomes more prone to further hospitalisations with rapid deterioration, resulting in a “snowball effect.” Moreover, previous predictive models to identify high-impact users have performed poorly due to lack of understanding of natural history of adverse events [42]. Sequence analysis provides common linkages of causes of readmission and insight into course of deteriorating events in the life of the patient.

The sequence analysis provided multiple benefits when applied to the hospital administrative data as compared to previous analysis of causes of readmission. In the previous studies, causes of readmissions were simply compiled for a selected cohort of patients [9]. Instead of simple analysis that reveals common causes of readmissions, sequence analysis provides additional information on the timing of the occurrence of events [17]. The commonest pattern of readmissions was identified for high-impact users. For all patients with emergency readmissions, the categories of causes of readmission were colour-coded and all sequences of readmissions were visualised [18]. In addition, the package can also run various common statistical tests between groups to assess association of various factors [18]. It has the ability to demarcate groups based on independent variables like sex, age, socioeconomic index, and so on [18]. For a given time-frame, the common causes of readmission and their subsequences can be investigated [18]. The program has the ability to run the analysis on a big data sample and for a large number of coded categories of readmissions [18].

The sequence analysis of hospital administrative data had certain limitations. Firstly, the identification of comorbidities was based on the secondary diagnoses that are variably recorded in administrative data [43]. We have tried to use all possible codes that define the condition to include most cases accurately. Secondly, the data did not include information on the pathological severity of stroke, which is also an important determinant of patient's morbidity [44]. Thirdly, selection of cases in a retrospective cohort study may lead to a degree of selection bias. Fourthly, the analysis was based on the causes of readmissions diagnosed in the hospital and may not provide information on preceding events that occurred in the community. The patient may have had treatment for chest infection in the hospital which could have been a result of a fall in the community. Fifthly, the sequence analysis only assesses a single type of repeated observations in the administrative data. In order to study a sequence of procedures, a separate analysis needs to be carried out. Sixthly, the patient follow-up was approximately and minimally of 4 years. The exact follow-up period is hard to calculate in the administrative data because the information for each hospital episode is recorded according to the National Health Service financial year every 1st of April.

## 5. Conclusions

Sequence analysis was effectively performed on hospital administrative data to study chronological order of causes of readmissions in the cerebrovascular conditions. Common and distinguishable subsequences of causes of readmissions among the high-impact users were identified that can be potentially avoided by targeting and placing appropriate resources in the community.

## Supplementary Material

The supplementary material consists of ICD-10 codes for various medical conditions, which were then combined to form diagnostic categories. The grouping was based on related pathologies and similar management pathways so that a single modifiable factor in the treatment algorithm can be identified to avoid the category of readmissions.

## Figures and Tables

**Table 1:** Characteristics of subgroups in the patients with cerebrovascular conditions.

Associated covariates	Ischaemic stroke				Intracranial haemorrhage				TIA			
	Low-impact (*n* = 25758)	Short-term high-impact (*n* = 7269)	Chronic high-impact (*n* = 1181)	*p* value	Low-impact (*n* = 1967)	Short-term high-impact (*n* = 548)	Chronic high-impact (*n* = 90)	*p* value	Low-impact (*n* = 15768)	Short-term high-impact (*n* = 4134)	Chronic high-impact (*n* = 647)	*p* value
Age (mean ± SD)	71.1 (13.7)	75.3 (12.1)	77.1 (11.5)	<0.01	69.9 (17.0)	73.4 (15.8)	76.9 (14.6)	<0.01	70.7 (13.7)	76.9 (11.8)	78.9 (11.7)	<0.01
Sex (male)	53.0%	49.2%	50.9%	<0.01	62.8%	62.7%	66.6%	0.76	50.0%	45.6%	46.2%	<0.01
Living alone	13.2%	17.6%	15.4%	<0.01	10.3%	13.3%	13.3%	0.10	9.6%	15.4%	13.9%	<0.01
Charlson comorbidity score > 1	36.7%	47.6%	57.6%	<0.01	34.6%	43.9%	52.2%	<0.01	33.5%	47.2%	55.2%	<0.01
Carstairs socioeconomic quintile (mean ± SD)	2.9 (1.4)	3.2 (1.4)	3.2 (1.4)	<0.01	2.9 (1.4)	3.2 (1.3)	3.1 (1.3)	<0.01	2.9 (1.3)	3.1 (1.3)	3.2 (1.3)	<0.01
Index stroke length of stay (mean ± SD)	12.9 (19.7)	18.5 (25.4)	21.3 (30.4)	<0.01	9.9 (15.4)	14.2 (22.9)	15.9 (20.9)	<0.01	2.5 (5.3)	3.99 (7.2)	5.49 (12.0)	<0.01
Number of Hospital acquired complications (mean ± SD)	0.3 (0.6)	0.4 (0.6)	0.5 (0.7)	<0.01	0.3 (0.6)	0.6 (0.7)	0.7 (0.9)	<0.01	0.2 (0.4)	0.34 (0.5)	0.41 (0.6)	<0.01
Dementia	3.5%	3.9%	6.2%	<0.01	7.0%	8.7%	10.0%	0.27	4.5%	6.1%	11.1%	<0.01
Renal failure	0.8%	1.3%	2.7%	<0.01	1.0%	1.4%	1.1%	0.68	0.5%	0.9%	1.0%	<0.01
Ischaemic heart disease	5.7%	8.3%	9.8%	<0.01	4.4%	6.0%	6.7%	0.22	5.9%	10.3%	11.7%	<0.01
Atrial fibrillation	21.9%	26.6%	31.7%	<0.01	15.6%	19.3%	20.0%	0.07	14.7%	22.4%	25.9%	<0.01
Anaemia	2.0%	2.7%	3.3%	<0.01	2.2%	2.7%	1.1%	0.56	1.7%	3.3%	4.3%	<0.01
Epilepsy	2.2%	3.9%	6.3%	<0.01	5.9%	8.9%	14.4%	<0.01	1.8%	3.3%	5.6%	<0.01
Hearing loss	1.7%	2.2%	2.5%	<0.01	2.0%	2.0%	1.1%	0.82	1.3%	2.3%	1.4%	<0.01
Vision loss	1.3%	2.1%	2.6%	<0.01	1.4%	1.8%	0.0%	0.39	1.1%	1.7%	2.0%	0.01
Paralysis	3.8%	4.6%	4.1%	<0.01	3.9%	4.9%	5.6%	0.67	1.7%	3.0%	3.1%	<0.01
Previous stroke	9.6%	16.7%	22.3%	<0.01	8.7%	15.9%	14.4%	<0.01	12.6%	23.9%	30.1%	<0.01
Bowel or urinary incontinence	2.9%	3.9%	6.1%	<0.01	2.4%	3.5%	5.6%	0.09	0.8%	1.3%	1.4%	<0.01
Mental health disorders	7.2%	9.2%	11.3%	<0.01	10.3%	13.7%	18.9%	<0.01	7.9%	10.7%	14.1%	<0.01
Use of thrombolysis	6.3%	5.9%	4.7%	0.01	—	—	—		—	—	—	
CNS procedures	0.8%	0.7%	0.3%	<0.01	18.7%	14.1%	10.0%	<0.01	0.6%	0.3%	0.1%	0.02
Other procedures	1.4%	1.9%	3.1%	<0.01	2.44%	4.01%	3.33%	0.13	0.29%	0.31%	0.46%	0.72
Discharge to nursing home	5.1%	5.9%	8.9%	<0.01	3.05%	3.65%	7.78%	0.04	0.96%	1.50%	2.32%	<0.01

**Table 2:** Five common causes of emergency readmissions per year for cerebrovascular diseases.

Ischaemic stroke			
Year 2011 (*n* = 21551)	Year 2012 (*n* = 25308)	Year 2013 (*n* = 21628)	Year 2014 (*n* = 19793)
Ischaemic stroke (15.29%)	Chest infection (18.54%)	Chest infection (20.83%)	Chest infection (22.30%)
Chest infection (14.37%)	Urine infection and urological conditions (14.53%)	Urine infection and urological conditions (16.04%)	Urine infection and urological conditions (16.55%)
External injuries (12.25%)	External injuries (12.36%)	External injuries (12.67%)	External injuries (12.57%)
Urine infection and urological conditions (12.04%)	Ambulatory conditions (11.32%)	Ambulatory conditions (10.43%)	Ischaemic stroke (10.59%)
Ambulatory conditions (10.72%)	Ischaemic stroke (9.49%)	Ischaemic stroke (8.49%)	Ambulatory conditions (7.85%)

Intracranial haemorrhage			
Year 2011 (*n* = 1734)	Year 2012 (*n* = 1768)	Year 2013 (*n* = 1433)	Year 2014 (*n* = 1312)

External injuries (16.61%)	Chest infection (21.32%)	Chest infection (23.03%)	Chest infection (25.91%)
Urine infection and urological conditions (12.86%)	External injuries (16.06%)	External injuries (15.07%)	Urine infection and urological conditions (15.55%)
Chest infection (11.88%)	Urine infection and urological conditions (12.78%)	Urine infection and urological conditions (14.10%)	External injuries (13.19%)
Dementia (10.09%)	Ambulatory conditions (7.75%)	Ambulatory conditions (7.82%)	Ambulatory conditions (8.31%)
Ambulatory conditions (7.50%)	Epilepsy/seizure (6.17%)	Epilepsy/seizure (5.72%)	Epilepsy/seizure (4.19%)

TIA			
Year 2011 (*n* = 12087)	Year 2012 (*n* = 15055)	Year 2013 (*n* = 13091)	Year 2014 (*n* = 11594)

Chest infection (14.46%)	Chest infection (18.82%)	Chest infection (19.33%)	Chest infection (21.16%)
External injuries (14.02%)	Urine infection and urological conditions (13.91%)	Urine infection and urological conditions (14.40%)	Urine infection and urological conditions (14.67%)
Ischaemic stroke (13.51%)	External injuries (12.89%)	External injuries (13.85%)	External injuries (13.33%)
Urine infection and urological conditions (12.29%)	Ambulatory conditions (11.29%)	Ambulatory conditions (12.10%)	Ambulatory conditions (11.56%)
Ambulatory conditions (11.92%)	Ischaemic stroke (9.64%)	Ischaemic stroke (8.88%)	Ischaemic stroke (8.00%)

**Table 3:** Most common subsequences in the subgroups of cerebrovascular conditions (urine infection and urological conditions [URO], respiratory tract infection [RTI], external injuries [INJ], ambulatory conditions [AMB], dementia [DEM], cerebrovascular conditions [IS], and epilepsy/seizure [EPI]).

Most common subsequences	Low-impact users (*n* [%])	Short-term high-impact users (*n* [%])	Chronic high-impact users (*n* [%])	*p* value
*Ischaemic stroke*	*n* = 11535	*n* = 7129	*n* = 1149	

URO-RTI	215 [1.87]	584 [8.2]	102 [8.9]	<0.01
RTI-URO	126 [1.1]	470 [6.6]	75 [6.6]	<0.01
INJ-URO	196 [1.7]	570 [8.1]	79 [6.9]	<0.01
RTI-AMB	126 [1.1]	377 [5.3]	88 [7.7]	<0.01
AMB-RTI	184 [1.6]	441 [6.2]	102 [8.9]	<0.01
AMB-URO	69 [0.6]	313 [4.4]	64 [5.6]	<0.01
URO-AMB	81 [0.7]	292 [4.2]	74 [6.5]	<0.01
URO-AMB	81 [0.7]	292 [4.2]	70 [6.1]	<0.01
INJ-RTI	207 [1.8]	491 [6.9]	74 [6.5]	<0.01
URO-DEM	69 [0.6]	242 [3.4]	43 [3.8]	<0.01

*Intracranial haemorrhage*	*n* = 798	*n* = 532	*n* = 83	

DEM-INJ	3 [0.4]	12 [5.4]	6 [7.2]	<0.01
RTI-DEM	3 [0.4]	5 [2.1]	7 [8.4]	<0.01
DEM-DEM-INJ	1 [0.1]	16 [3.0]	5 [6.0]	<0.01
DEM-URO	5 [0.6]	7 [3.2]	7 [8.4]	<0.01
INJ-URO	20 [2.5]	46 [8.6]	7 [8.4]	<0.01
EPI-INJ	2 [0.2]	17 [3.2]	5 [6.0]	<0.01
URO-INJ	18 [1.3]	35 [6.6]	5 [6.0]	<0.01
URO-RTI	17 [2.2]	42 [7.9]	6 [7.2]	<0.01
INJ-RTI	23 [2.9]	36 [6.8]	11 [13.2]	<0.01
RTI-INJ	7 [0.9]	27 [5.1]	4 [4.8]	<0.01

*TIA*	*n* = 6905	*n* = 4046	*n* = 624	

INJ-URO	110 [1.6]	360 [8.9]	43 [6.9]	<0.01
URO-RTI	103 [1.5]	320 [7.9]	54 [8.8]	<0.01
URO-INJ	103 [1.5]	320 [7.9]	46 [7.4]	<0.01
AMB-URO	48 [0.7]	214 [5.3]	47 [7.5]	<0.01
AMB-RTI	110 [1.6]	262 [6.5]	62 [10.0]	<0.01
URO-AMB	41 [0.6]	182 [4.5]	44 [7.1]	<0.01
RTI-AMB	97 [1.4]	234 [5.8]	57 [9.1]	<0.01
DEM-URO	48 [0.7]	178 [4.4]	43 [6.9]	<0.01
RTI-URO	76 [1.1]	218 [5.4]	44 [7.1]	<0.01
IS-URO	83 [1.2]	226 [5.6]	26 [4.2]	<0.01

**Table 4:** Comparison of common subsequences among groups of high-impact users in the cerebrovascular conditions (urine infection and urological conditions [URO], respiratory tract infection [RTI], external injuries [INJ], ambulatory conditions [AMB], dementia [DEM], ischaemic stroke [IS], epilepsy/seizure [EPI], ischaemic heart disease [IHD], other infections and inflammation of organs/skin [INF], bleeding complications [BLEED], and metabolic and nutritional disorders [MET]).

Common subsequences of readmissions among high-impact users	Short-term high-impact (%)	Chronic high-impact (%)	*p* value
*Ischaemic stroke*	*n* = 7129	*n* = 1149	

AMB-IHD-IHD-AMB	78 [1.1]	31 [2.7]	0.014
AMB-RTI-RTI-AMB	99 [1.4]	34 [3.1]	0.017
AMB-RTI	427 [6.0]	102 [8.9]	0.012
RTI-AMB-RTI	71 [1.0]	25 [2.2]	<0.01
RTI-AMB-RTI-AMB	142 [2.1]	42 [3.7]	<0.01
RTI-AMB	370 [5.2]	87 [7.7]	<0.01
EPI-RTI	128 [1.8]	37 [3.2]	<0.01
URO-AMB	299 [4.2]	69 [6.1]	<0.01

*Intracranial haemorrhage*	*n* = 532	*n* = 83	

RTI-MET	5 [0.9]	5 [6.0]	<0.01
RTI-DEM	11 [2.1]	7 [8.4]	<0.01
MET-DEM	3 [0.6]	4 [4.8]	<0.01
DEM-EPI	6 [1.1]	5 [6.0]	<0.01
INJ-INJ-RTI	11 [2.1]	6 [7.2]	0.021
DEM-URO	17 [3.2]	7 [8.4]	0.046
INJ-RTI	36 [6.7]	11 [13.2]	0.064
IHD-AMB	11 [2.1]	5 [6.0]	0.082
BLEED-AMB	4 [0.7]	3 [3.6]	0.083

*TIA*	*n* = 4046	*n* = 624	

INJ-URO	65 [1.6]	56 [8.9]	<0.01
URO-RTI	61 [1.5]	49 [7.9]	<0.01
URO-INJ	61 [1.5]	49 [7.9]	<0.01
AMB-URO	28 [0.7]	33 [5.3]	<0.01
AMB-RTI	65 [1.6]	41 [6.5]	<0.01
RTI-AMB	238 [5.9]	57 [9.1]	<0.01
AMB-INJ-INJ-AMB	32 [0.8]	14 [2.2]	<0.01
INF-AMB	73 [1.8]	22 [3.6]	<0.01
URO-AMB	182 [4.5]	44 [7.1]	<0.01
DEM-URO	178 [4.4]	43 [6.9]	<0.01
