# Supplementary material for: Sequence Analysis of Long-Term Readmissions among High-Impact Users of Cerebrovascular Patients
Source: Stroke Res Treat. 2017 May 16;2017:7062146. doi: 10.1155/2017/7062146 (PMC5448070; doi:10.1155/2017/7062146)
Supplement: Supplementary file 1 — The supplementary material consists of ICD-10 codes for various medical conditions, which were then combined to form diagnostic categories. The grouping was based on related pathologies and similar management pathways so that a single modifiable factor in the treatment algorithm can be identified to avoid the category of readmissions. [file 7062146.f1.docx]

ICD-10 codes used for the identification of the common causes of emergency readmission

Gastro-intestinal infections: 'A047','A020','A029','A039','A045','A047','A048','A049','A052','A054',

'A059','A080','A081','A083','A084','A090','A099','A09X','B378'

Respiratory tract infections 'J00X','J010','J019','J028','J029','J039','J040','J041','J042','J051',

'J060','J069','J100','J101','J108','J110','J111','J118','J120','J121',

'J122','J129','J128','J129','J13X','J14X','J150','J151','J152','J153',

'J154','J155','J156','J157','J158','J159','J160','J168','J170','J171',

'J172','J173','J178','J180','J181','J182','J188','J189','J200','J201',

'J202','J203','J204','J205','J206','J207','J208','J209','J210','J218',

'J219','J22','J22X','J440','J441','J690','J691','J698','J702','J704',

'J708','J709','J850','J851','J852','J960','J969','J981','J982','J985',

'J986','R092','J00','J028','J029','J040','J041','J042','J050','J051',

'J060','J068','J069','J170','B380','B450','B460','B440','B441','B420',

'J998','B390','B392','B400','B441','B420','J998','B390','B392','B400',

'B402','B59','B012','B019','J40X','J411','J42X','J432','J349','J440',

'J441','J448','J449','J450','J459','J46X','J47X','J860','J869','J90X'

Gastro-intestinal bleeding

'K920','K920','K921','K922','K928','K929','R040','R31X'

Cardiac arrhythmias

'I470','I471','I472','I479','I490','I491','I493','I494','I495','I498',

'I499','I48X'

Urine infection and urological conditions 'N170','N171','N178','N179','N10','N12','N151','N159','N158','N160',

'N288','N300','N302','N304','N303','N308','N309','N340','N341','N342',

'N370','N390','N410','N450','N459','N47','N511','A540','A560','A562',

'B374','N310','N311','N391','N394','N398','N399','N312','N319','N318',

'N393','R31','R32','R33','R34','R33X','R200','R201','R202','R203',

'R208','R21X','R220','R221','R222','R223','R224','R227','R229','R230',

'R231','R232','R233','R234','R238','R251','R252','R253','R258','R260',

'R261','R262','R263','R268','R270','R278','R290','R293','R296','R298',

'R300','R309','R31X','R32X','R33X','R34X','R35X','R36X','R390','R391',

'R398'

Cerebrovascular conditions 'G450','G451','G452','G453','G454','G458','G459','G460','G461','G462',

'G463','G464','G465','G466','G467','G468','H930','H340','H341','H342',

'H348','H349','I630','I631','I632','I633','I634','I635','I636','I638',

'I639','I64','I64X','I650','I651','I652','I653','I658','I659','I660',

'I661','I662','I663','I664','I668','I669','I670','I671','I672','I677',

'I678','I679','I681','I682','I693','I694','I698'

Ischaemic heart disease 'I200','I201','I208','I209','I210','I211','I212','I213','I214','I219',

'I220','I221','I228','I229','I230','I231','I232','I233','I234','I235',

'I236','I238','I240','I241','I248','I249','I251','I252','I253',

'I254','I255','I258','I259'

Pulmonary embolism and deep venous thrombosis

'I260','I269'

Dementia

'F000','F001','F002','F010','F011','F012','F013','F018','F019','F020','F021','F022','F023','F024','F028','F03X',

'G300','G301','G308','G309','F04X','F060','F062','F063','F064','F067','F068','F069','F070','F072','F078','F079',

'F701','F709','F711','F719','F799','F09X',

'R400','R401','R402','R410','R411','R413','R418','R42X','R438','R440','R441','R442','R443','R448','R450',

'R451','R452','R454','R455','R456','R457','R458','R462','R464','R468',

'R410','R54X'

Non-traumatic intra-cerebral haemorrhage

'I600','I601','I602','I603','I604','I605','I606','I607','I608','I609',

'I690','I610','I611','I612','I613','I614','I615','I616','I618','I619',

'I620','I621','I629'

Peripheral vascular disease

'I700','I7000','I701','I7010','I702','I7020','I7021','I708','I7080',

'I709','I7090','I7091','I730','I731','I738','I739','I771','I772','I740',

'I741','I742','I743','I744','I745','I748','I749'

External injuries (including fractures)

'S000','S001','S002','S003','S004','S005','S006','S007','S008','S009',

'S010','S011','S012','S013','S014','S015','S016','S017','S018','S019',

'S020','S0200','S021','S0210','S022','S0220','S0221','S0230','S0231',

'S0240','S0250','S0260','S0270','S0280','S0290','S0600','S0620','S0630',

'S0641','S0640','S0650','S0680','S0660','S0650','S0651','S0680',

'S0681','S0690','S0691','S023','S024','S025','S026','S027','S028','S029',

'S030','S031','S032','S3250','S033','S034','S035','S040','S041','S042',

'S043','S044','S045','S046','S047','S048','S049','S050','S051','S052',

'S053','S054','S055','S056','S057','S058','S059','S060','S061','S062',

'S063','S064','S065','S066','S067','S068','S069','S070','S071','S078',

'S079','S080','S081','S088','S089','S090','S091','S092','S079','S080',

'S081','S088','S089','S090','S091','S092','S097','S098','S099','S100',

'S101','S107','S108','S109','S110','S111','S112','S118','S119','S120',

'S121','S122','S127','S128','S129','S130','S131','S132','S133','S134',

'S135','S136','S140','S141','S142','S143','S144','S145','S146','S150',

'S150','S151','S152','S153','S157','S158','S159','S16','S170','S178',

'S179','S18','S197','S198','S199','S1200','S1210','S1220','S1270',

'S1290','S2220','S2230','S2240','S2250','S2680','S2690','S2700','S2710',

'S2720','S2210','S2730','S200','S201','S202','S203','S204','S205','S206',

'S207','S208','S21','S210','S211','S212','S217','S218','S219','S220',

'S221','S222','S223','S224','S225','S228','S229','S230','S231','S232',

'S233','S234','S235','S240','S241','S242','S243','S244','S245','S246',

'S250','S251','S252','S253','S254','S255','S257','S258','S259','S260',

'S268','S269','S270','S271','S272','S273','S274','S275','S276','S277',

'S278','S279','S280','S281','S290','S297','S298','S299','S400','S407',

'S408','S409','S410','S411','S417','S418','S420','S421','S4200','S4210',

'S4220','S4230','S4231','S4240','S4241','S4270','S4280','S4290','S422',

'S423','S424','S423','S424','S425','S426','S427','S428','S429','S430',

'S431','S432','S433','S434','S435','S436','S437','S440','S441','S442',

'S443','S444','S445','S446','S447','S448','S449','S450','S451','S452',

'S453','S454','S455','S456','S457','S458','S459','S460','S461','S462',

'S463','S467','S468','S469','S47','S480','S481','S489','S497','S498',

'S499','T68X','T733','T741','T751','T753','J60X','J61X','J64X','J679',

'J680','J690','J698','J700','J703','J704','J930','J931','J938','J939',

'J942','L560','L561','L562','L569','L570','L574','L578','L579',

'L581','L589','L598','K721','K729','M660','M6606','M6623','M6624',

'M6634','M6641','M6653','S300','S301','S302','S3200','S3220','S3230',

'S3240','S3280','S3250','S3600','S3601','S3601','S3650','S3660','S3671',

'S3680','S3700','S3720','S3730','S3790','S307','S308','S309','S310',

'S311','S312','S313','S314','S315','S316','S317','S318','S320','S321',

'S322','S323','S324','S325','S326','S327','S328','S330','S331','S332',

'S333','S334','S335','S336','S337','S340','S341','S342','S343','S344',

'S345','S346','S347','S348','S350','S351','S352','S353','S354','S355',

'S356','S357','S358','S359','S360','S361','S362','S363','S364','S365',

'S367','S368','S369','S370','S371','S372','S373','S374','S375','S376',

'S377','S378','S380','S381','S382','S383','S390','S396','S397','S398',

'S399','S500','S501','S507','S508','S509','S510','S517','S518','S519',

'S520','S521','S522','S523','S524','S525','S526','S5200','S5201','S5210',

'S5220','S5221','S5230','S5240','S5250','S5251','S5260','S5261','S5270',

'S5280','S5290','S527','S528','S529','S530','S531','S532','S533','S534',

'S540','S541','S542','S543','S547','S548','S549','S550','S551','S557',

'S558','S559','S560','S561','S562','S563','S564','S565','S566','S567',

'S568','S559','S560','S561','S562','S563','S564','S565','S566','S567',

'S568','S570','S578','S579','S580','S581','S589','S597','S598','S599'

'S600','S601','S602','S6200','S6210','S6220','S6230','S6231','S6240',

'S6250','S6251','S6260','S6261','S6270','S6280','S607','S608','S609',

'S610','S611','S617','S618','S619','S620','S621','S622','S623','S624',

'S625','S626','S627','S628','S630','S631','S632','S633','S634','S635',

'S636','S637','S640','S641','S642','S643','S644','S647','S648','S649',

'S650','S651','S652','S653','S654','S655','S656','S657','S658','S659',

'S660','S661','S662','S663','S664','S665','S666','S667','S668','S669',

'S670','S678','S680','S681','S682','S683','S684','S688','S689','S697',

'S698','S699''S700','S701','S707','S708','S710','S718','S720','S721',

'S722','S723','S724','S727','S728','S729','S730','S731','S740','S741',

'S742','S747','S749','S750','S751','S752','S757','S758','S759','S760',

'S761','S762','S763','S764','S767','S770','S771','S772','S780','S781',

'S789','S797','S798','S799','S7200','S7201','S7210','S7211','S7220',

'S7230','S7240','S7241','S7270','S7280','S7290','S800','S801','S807',

'S808','S809','S810','S817','S818','S819','S820','S821','S822',

'S8200','S8201','S8210','S8211','S8220','S8221','S8230','S8231','S8240',

'S8250','S8251','S8260','S8261','S8270','S8271','S8280','S8281',

'S823','S824','S825','S826','S827','S828','S829','S830','S831','S832',

'S833','S834','S835','S836','S837','S840','S841','S842','S847','S848',

'S849','S850','S851','S852','S853','S854','S855','S856','S857','S858',

'S859','S860','S861','S862','S863','S867','S868','S869','S870','S878',

'S880','S881','S889','S900','S901','S902','S903','S907','S908','S909',

'S910','S911','S912','S913','S917','S920','S921','S922','S923','S924',

'S925','S926','S927','S929','S930','S931','S932','S933','S934','S935',

'S936','S940','S941','S942','S943','S947','S948','S949','S950','S951',

'S952','S957','S958','S959','S960','S961','S962','S967','S968','S969',

'S970','S971','S978','S980','S981','S982','S983','S984','S997','S998',

'S999','S9200','S9210','S9220','S9230','S9240','S9241','S9250','S9251',

'S9270','T000','T001','T002','T003','T006','T008','T009','T010','T011',

'T012','T013','T014','T016','T018','T019','T020','T021','T022','T0200',

'T0210','T0220','T0240','T0250','T0280','T023','T024','T025','T026',

'T027','T028','T029','T030','T031','T032','T033','T034','T038','T039',

'T040','T041','T042','T043','T044','T047','T048','T049','T050','T051',

'T052','T053','T054','T055','T056','T058','T059','T060','T061','T062',

'T063','T064','T065','T068','T07','T08','T090','T091','T092','T093',

'T094','T095','T096','T098','T099','T10','T110','T111','T112','T113',

'T114','T115','T116','T117','T118','T119','T12','T130','T131','T132',

'T133’,'T134','T135','T136','T138','T139','T140','T141','T142','T143',

'T144','T145','T146','T147','T148','T149','T150','T151','T158','T159',

'T16','T170','T171','T172','T173','T174','T175','T178','T179','T180',

'T181','T182','T183','T184','T185','T186','T187','T188','T189','T190',

'T191','T192','T193','T198','T199','T200','T201','T202','T203','T204',

'T205','T206','T207','T210','T211','T212','T213','T214','T215','T216',

'T217','T220','T221','T222','T223','T224','T225','T226','T227','T230',

'T231','T232','T233','T234','T235','T236','T237','T240','T241','T242',

'T243','T244','T245','T246','T247','T250','T251','T252','T253',

'T254','T255','T256','T257','T330','T331','T332','T333','T334','T335',

'T336','T337','T338','T339','T340','T341','T342',

'T343','T344','T345','T346','T347','T348','T349','T350','T351','T352',

'T353','T354','T355','T356','T357','T360','T363','T365','T369','T374',

'T378','T381','T383','T390','T391','T393','T398','T399','T401',

'T402','T403','T404','T405','T406','T415','T420','T421','T422','T424',

'T425','T426','T427','T428','T430','T432','T433',

'T434','T435','T436','T438','T443','T446','T447','T449','T450','T451',

'T452','T455','T460','T461','T462','T463','T464',

'T465','T466','T469','T470','T471','T474','T490','T497','T500','T501',

'T502','T503','T504','T506','T508','T509','T510',

'T518','T549','T568','T58X','T597','T598',

'R42X','R55X','M800','M801','M802','M803','M804','M805','M806','M807',

'M808','M809','R55','W00','W01','W02','W03','W04','W05','W06'

'W06','W07','W08','W09','W10','W17','W18','W19','S720','S721','S722',

'S723','S724','S727','S728','S729'

Iatrogenic complications

'T604','T608','T609','T629','T634','T658',

'T780','T781','T782','T783','T784','T793','T795','T796','T797','T801',

'T802','T809','T810','T811','T812','T813','T814','T815','T816','T817',

'T818','T819','T820','T821','T822','T823','T824','T825','T826','T827',

'T828','T829','T830','T831','T832','T833','T834','T835','T836','T838',

'T839','T840','T841','T842','T843','T844','T845','T846','T847','T848',

'T849','T850','T851','T852','T853','T854','T855','T856','T857','T858',

'T859','T860','T861','T864','T868','T873','T874','T875','T876','T884',

'T885','T886','T887','T888','T889','T602','Y600','Y601','Y602','Y603',

'Y604','Y605','Y606','Y607','Y608','Y609','Y610','Y611','Y612','Y613',

'Y614','Y615','Y616','Y617','Y618','Y619','Y620','Y621','Y622','Y623',

'Y624','Y625','Y626','Y627','Y628','Y629','Y630','Y631',

'Y632','Y633','Y634','Y635','Y636','Y637','Y638','Y639','Y640','Y641',

'Y648','Y649','Y650','Y651','Y652','Y653','Y654','Y655',

'Y656','Y658','Y66','Y69','Y70','Y71','Y74','Y75','Y880','Y881','Y882',

'Y883','T810','T811','T812','T813','T814','T815','T816',

'T817','T818','T819','T820','T821','T822','T823','T824','T825','T826',

'T827','T828','T829','T830','T831','T832','T833','T834',

'T835','T836','T837','T838','T839','T840','T841','T850','T851','T857',

'T858','T859','T876','T880','T881','T882','T883','T884','T885',

'T886','T887','T888','T889','T870','T871','T872','T873','T874','T875',

'T876','N141','N142','N151','N152','J950','J955','J958',

'G938','H590','H598','O860','O901','O908','O909','G971','G972','G978'

Metabolic and nutritional disorders

'D500','D508','D509','D510','D518','D519','D529','D531','D539','D501',

'D610','D611','D619','D62X','D630','D638','D640','D648','D649','E209',

'E210','E211','E212','E213','E41X','E43X','E46X','E512','E519','E531',

'E538','E559','E561','E611','E612','E638','E662','E668','E669','E662','E668','E669','E721','E752','E780','E781','E785','E801','E806','E831',

'E833','E834','E835','E86X','E870','E871','E872','E873','E875','E876',

'E877','E878','E880','E888','E889','E890','E892',

'E893','E120','E121','E129','E15','E870',

'E871','E872','E873','E874','E875','E876','E877','E878','R730','R739'

Ambulatory conditions

'E100','E101','E102','E103','E104','E105','E106','E109','E110','E111',

'E112','E113','E114','E115','E116','E118','E119','E130','E131','E139',

'E140','E141','E143','E145','E149','E160','E161','E162','E169',

'I500','I501','I509','J432','J349','J440','J441','J448','J449',

'J450','J459','J46X','J47X','R000','R001','R002','R008','R011','R02X',

'R030','R031','R040','R042','R048','R05X','R060','R061','R062',

'R063','R064','R065','R066','R068','R070','R071','R072','R073','R074',

'R090','R091','R092','R098','R070','R071','R072','R073','R074'

Delirium

'F050','F051','F058','F059','R410','F100','F101','F102','F103','F104',

'F105','F106','F107','F109','F110','F111','F112','F121','F125','F137',

'F141','F170','F171','F190','F191','F192','F194','F195'

Epilepsy and seizures 'G401','G402','G403','G404','G405','G406','G407','G408','G409','G410',

'G411','G412','G418','G419','R56','R560','R568', 'F068','F069',

Other infections/inflammation of internal organs and skin

'B023','B309','B022','B159','B179','B181','B182','B199','B258','B259',

'B440','B441','B448','B465','B509','B550','B789','B962','B99X','I330',

'B376','I400','I409','B332','B338','I800','II801','I802','I803','I808',

'I809','K20X','K210','K219','K290','K291','K292','K293','K294','K295',

'K296''K297','K298','K299','K30X','K800','K801','K802','K803','K804',

'K805','K808','K810','K811','K818','K819','K823','K828''K829','K830',

'K831,'K832','K833','K835','K838','K839','K850','K851','K852','K853',

'K858','K859','K85X','M0000','M0001','M0003','M0004','M0005','M0006',

'M0016','M0080','M0081','M0086','M0087','M009','M0090','M0091','M0092',

'M0093','M0094','M0095','M0096','M0097','M0290','M0295','M0296','M0320',

'N709','N710','N711','N719','N72X','N735','N736','N739','N750','N751',

'N762','N763','N764','N766','N768','K570','K571','K572','K573','K574',

'K575','K578','K579','K650','K658','K659','K660','K661','K668','K669',

'A46X','A490','A491','A492','A498','A662','A70X','B028','B029','B07X',

'B372','B373','B374','B379','B86X','B880','L011','L020','L021','L022',

'L023','L024','L028','L029','L030','L031','L032','L033','L038',

'L039','L040','L041','L042','L048','L050','L059','L080','L088','L089'
